# TNFα-induced altered miRNA expression links to NF-κB signaling pathway in endometriosis

**DOI:** 10.21203/rs.3.rs-2870585/v1

**Published:** 2023-05-04

**Authors:** Saswati Banerjee, Wei Xu, Aaron Doctor, Adel Driss, Ceana Nezhat, Neil Sidell, Robert N Taylor, Winston E Thompson, Indrajit Chowdhury

**Affiliations:** Morehouse School of Medicine; Morehouse School of Medicine; Morehouse School of Medicine; Morehouse School of Medicine; Nezhat Medical Center; Emory University School of Medicine; University at Buffalo; Morehouse School of Medicine; Morehouse School of Medicine

**Keywords:** Endometriosis, TNFα, miRNA, signaling

## Abstract

Endometriosis is a common gynecological inflammatory disorder characterized by immune system dysregulation, which is involved in lesion initiation and progression. Studies have demonstrated that several cytokines are associated with the evolution of endometriosis, including tumor necrosis factor-α (TNFα). TNFα is a non-glycosylated cytokine protein with potent inflammatory, cytotoxic, and angiogenic potential. In the current study, we examined the ability of TNFα to induce dysregulation of microRNAs (miRNAs) linked to NFkB-signaling pathways, thus contributing to the pathogenesis of endometriosis. Using RT-QPCR, the expression of several miRNAs were quantified in primary cells derived from eutopic endometrium of endometriosis subjects (EESC) and normal endometrial stromal cells (NESC) and also TNFα treated NESCs. The phosphorylation of the pro-inflammatory molecule NF-κB and the candidates of the survival pathways PI3K, AKT and ERK was measured by westernblot analysis. The elevated secretion of TNFα in EESCs downregulates the expression level of several miRNAs significantly (p < 0.05) in EESCs compared to NESC. Also treatment of NESCs with exogenous TNFα significantly reduced the expression of miRNAs in a dose-dependent manner to levels similar to EESCs. In addition, TNFα significantly increased the phosphorylation of the PI3K, AKT, ERK, and NF-κB signaling pathways. Notably, treatment with curcumin (CUR, diferuloylmethane), an anti-inflammatory polyphenol, significantly increased the expression of dysregulated miRNAs in EESC in a dose-dependent manner. Our findings demonstrate that TNFα is upregulated in EESCs, which subsequently dysregulates the expression of miRNAs, contributing to the pathophysiology of endometriotic cells. CUR effectively inhibits the expression of TNFα, subsequently altering miRNA levels and suppresses the phosphorylation of AKT, ERK, and NF-κB.

## Background

Endometriosis is a benign, estrogen-dependent inflammatory disease characterized by the presence of endometrial tissue (specifically glands and stroma) outside of the uterus [1] [2] [3]. The exact causes of endometriosis remain unknown. The theory of retrograde menstruation, an efflux of menstrual blood and cells via the fallopian tubes to extrauterine sites, is considered an important origin of endometriosis lesions [1] [3]. While 90% of reproductive-aged women experience retrograde menstruation, only 10% are diagnosed with endometriosis [4]. Therefore, in addition to retrograde menstruation, other factors are likely involved in the pathogenesis of endometriosis, including hormonal imbalance, metabolic environment, epithelial-mesenchymal transition, altered immunity, and abnormal regulation of inflammation in endometrial cells (ECs) of genetically susceptible women [3]. In the peritoneal cavity, resident or recruited immune cells secrete excessive levels of proinflammatory cytokines that trigger inflammatory reactions in endometrial cells and promote lesion development and disease progression [1] [5] [6] [7].

Cytokines are small, soluble, diverse pleiotropic immunoregulatory signaling proteins with a short half life. Women with endometriosis have elevated levels of certain cytokines, including TNFα, that can stimulate EC proliferation, survival, migration, and adhesion to the peritoneal cavity, angiogenesis, and inflammation, which ultimately promote progression of the disease [8] [9] [10] [11] [12] [13] [14] [15] [16]. Cytokines, including TNFα, mediate their action through their receptors that activate a cascade of intracellular events, including nuclear factor-kappa B (NF-κB) signaling pathways [17] [8] [9] [10] [11]; [12] [13] [14] [15] [16]. NF-κB has been shown to orchestrate various physiological and pathophysiological responses of ECs and endometriosis [18] [19] [20] [21]) [22] [17]. Previous studies have demonstrated that women with endometriosis have increased NF-κB expression that regulates the expression of aberrant cytokines through autocrine self-amplifying cycles of cytokine release and NF-κB activation. These lead to amplification and maintenance of the proinflammatory local environment, promoting the survival and growth of ECs in endometriosis patients and reducing the clearance of retrogradely transported endometrial fragments [23] [24] [18] [19] [20] [21] [22].

Recent studies also demonstrated the aberrant dysregulation of microRNA (miR) expression in circulation as well as in ectopic and eutopic endometrium tissues of endometriotic patients [25] [26] [27] [28] [29] [30] [31] [27] [32] [33] [34] [35] [36] [37] [38] [37] [39] [40]. miRNAs are a large family of short, non-coding, single-stranded RNAs that are involved in the post-transcriptional regulation of cellular processes by binding to complementary sequences in the coding, 5’- or 3’- untranslated region (UTR) of target mRNAs that are subsequently silenced or degraded [41] [42] [43] [44] [45] [46]. Several pieces of evidence suggest that NF-κB signaling, is overactive in endometriotic lesions and plays a vital role in the onset, progression, and recurrence of endometriosis [47]. As important transcriptional regulators, miRNAs can modify many target genes involved in cytokine expression and the NF-κB signaling pathway via negative or positive feedback loops, and these have been identified as potentially robust biomarkers for endometriosis both in circulation and tissues [26] [27] [34] [48] [25] [41] [42] [40].

The TNFα dependent regulation of the expression of miRNAs associated with endometriosis in eutopic ECs is not well defined. Based on the proinflammatory nature of the disease, combined with the published data [18] [19] [22] [16] [41] [42] [49] and our comparative nanostring analysis of miRNAs (unpublished) between EESC and NESC, we aimed to analyze whether up-regulation of TNFα expression in the eutopic stromal cells of endometriotic patients induces the dysregulation of miRNAs linked to NF-kB-signaling pathways thus contributing to the pathogenesis of the disease. To evaluate this theory, the expression levels of proinflammatory and proangiogenic miRNAs were compared between the stromal cells of women with (EESC) and without endometriosis (NESC). Moreover, the effect of exogenous TNFα was tested on the expression of those selected miRNAs in NESC and whether their altered expressions are linked to the phosphorylation of NF-κB, PI3K, AKT, and/or ERK1/2 pathways. Our previous studies established that curcumin, a natural medicinal Asian herb with strong anti-inflammatory and antioxidant properties, attenuates proangiogenic and proinflammatory factors in human eutopic endometrial stromal cells through the NF-κB signaling pathway. In the current study, we further evaluated the effect of curcumin in altering the expression of proinflammatory miRNAs linked to the NF-κB signaling pathway. Taken together, we established that TNFα is upregulated in EESCs which subsequently increases the expression of proangiogenic and proinflammatory miRNAs, potentially contributing to the pathophysiology of endometriotic cells. We have determined that CUR effectively reduces the expression of TNFα and dysregulation of miRNA levels, and attenuate the phosphorylation status of PI3K, AKT, ERK, and NF-κB pathw,ays.

## Materials and methods

### Human subjects and tissue acquisition

The details about the source of primary endometrial stromal cells (ESCs) used in this study were described previously [16]. The current studies were approved by the institutional review boards of Emory University and Morehouse School of Medicine, Atlanta.

### Endometrial stromal cell (ESC) cultures

Primary endometrial stromal cells (ESCs) from human eutopic endometrial biopsies from women with (EESC) and without evidence of endometriosis (NESC) were prepared according to [50]. Cells (passages 3–5) were cultured and routinely maintained in Dulbecco’s Modified Eagle’s Medium/Ham’s Nutrient Mixture F-12 (DMEM/Ham’s F-12; Life Technologies, Inc.-BRL) supplemented with 12% fetal bovine serum (FBS; Thermo Fisher Scientific, Grand Island, NY, USA), 1% non-essential amino acids, 1% sodium pyruvate, and 1% penicillin-streptomycin (Penstrep, Sigma-Aldrich, St Louis, MO, USA), within a 5% CO_2_ atmosphere at 37 C in a humidified incubator. Cells were grown to 80% confluency in 100 mm plates (Corning, NY, USA). The culture media was replaced with low serum-containing media overnight before any experiments. After 24h, cells were treated or untreated in the DMEM/Ham’s F-12 medium supplemented with 0.4% FBS, 1% non-essential amino acids, 1% sodium pyruvate, and 1% Penstrep, and incubated at 37 C in a humidified incubator with 5% CO2 for 24h. Images of ESCs cultures were taken at 24 and 48 hours posttreated or untreated condition using an inverted phase contrast microscope. Unless specified differently, 20 random phase contrast images were acquired per well at 200× magnification.

### TNFα treatment of normal endometrial stromal cells (NESCs)

NESCs were grown up to 80% confluency in 100 mm plates as described above. Cells were serum-starved for 24 hours and then treated with TNFα (10 and 50 ng/ml, Sigma-Aldrich, USA) for 24 hours. The dose and time of treatment for TNFα are based on our unpublished work and published literature [51]. Cells were harvested for the estimation of total RNA and protein.

### Curcumin (CUR) treatment of normal and eutopic endometriotic stromal cells (NESCs, EESCs)

NESC and EESC cultures were grown to 80% confluency in 100 mm plates, as described above. Cells were treated with CUR (molecular weight 368.41, purity 99%, Sigma-Aldrich, USA) at a concentration of 5 and 10 μg/ml for 48h [16]. CUR was dissolved in dimethylsulfoxide (DMSO) and diluted to the desired concentrations in DMEM/Ham’s F-12 media with 0.4% serum-containing media followed by sterilization through 0.22μm membrane filtration. Cells were treated with the equivalent concentrations of DMSO added to the medium for the parallel vehicle control experiments. The final concentration of DMSO was less than 0.1%.

### Isolation of total RNA

Total RNA from NESC and EESC and corresponding curcumin or TNFα-treated ESCs was extracted using Qiagen miRNeasy Mini kit (Germantown, MD, USA) according to the manufacturer’s instructions. The quality of the extracted RNA was verified via absorbance measurements at wavelengths of 230, 260, and 280 nm using a spectrophotometer (NanoDrop, 2000; Thermo Fisher Scientific, Inc., Waltham, Massachusetts, USA). RNA 260/280 ratio of 1.9 or greater and 260/230 ratio of 1.8 or greater were used to obtain optimal results for the miR analysis.

### microRNA (miR) expression analysis

The RNA samples were transcribed using the miRCURY LNA RT kit (Germantown, MD, USA) according to the manufacturer’s protocol. Real-time PCR was performed using miRCURY LNA SYBR^®^ Green PCR Kit (Germantown, MD, USA) and LNA-enhanced and Tm-normalized miRNA primers from Qiagen on CFX connected Real-Time PCR Detection System (Bio-Rad Laboratories, Hercules, CA). All steps were performed according to the Qiagen MicroRNA assay protocol (Germantown, MD, USA). The relative expression of the gene was calculated using 2^−ΔΔCT^ methods with 5S rRNA (hsa) and U6 snRNA (hsa), as the reference miRs.

### Assessment of TNFα in secretion media

TNFα was measured in postculture media collected at 24 and 48 hours using Bio-Plex ProTM Human Cytokine, Chemokine, and Growth Factor Magnetic Bead-Based Assays (BioRad, Hercules, California, USA) coupled with the Luminex 200^™^ system (Austin, TX, USA) according to the manufacturer’s protocol. Samples were tested at a 1:2 dilution using optimal concentrations of standards and antibodies according to the manufacturer’s protocol.

### Western blot analysis

Total protein was extracted from different treatment conditions from untreated and treated NESC and EESC and subjected to one-dimensional gel electrophoresis and western blot (WB) analysis [52]. For one-dimensional gel electrophoresis, equal amounts of protein (25 μg) were applied to each lane. Primary antibodies were used as described in Table 1. Membranes were incubated with the appropriate secondary antibodies for 1 hour at room temperature, and protein-antibody complexes were visualized using SuperSignal^™^ West Pico detection reagent (Thermo fisher scientific, Waltham, MA) on an iBright^™^ FL1500 Imaging System (Thermo fisher scientific, Waltham, MA). Results of representative chemiluminescence were scanned and densitometrically analyzed using a Power Macintosh Computer (G3; Apple Computer, Cupertino, CA) equipped with a Scan Jet 6100C Scanner (Hewlett-Packard, Greeley, CO). Quantification of the scanned images was performed using NIH Image version 1.61 software (NIH, Bethesda, MD) (34).

## Statistical analysis

Data are expressed as mean ± SEM of three independent experiments. Statistical analysis was performed by one-way ANOVA using SPSS version 11.0 software (SPSS, Chicago, IL) to test the significance of differences in dose, duration, and interaction between dose and duration. Post-hoc corrections for multiple comparisons were done by Newman-Keuls’ test or unpaired Student’s t-test. Differences were considered significant at P ≤ 0.05. For miR expression analysis, fold change was calculated using a selected miR expression in a target sample relative to a control sample, normalized over a reference miR. The 2–ΔΔCt method was used and the ΔΔCt was calculated using the average of the control values. That generates multiple values close to 1 for the control and gives a standard error of the mean.

## Results

### EESC secrete higher concentrations of TNFα

**Figure 1:**
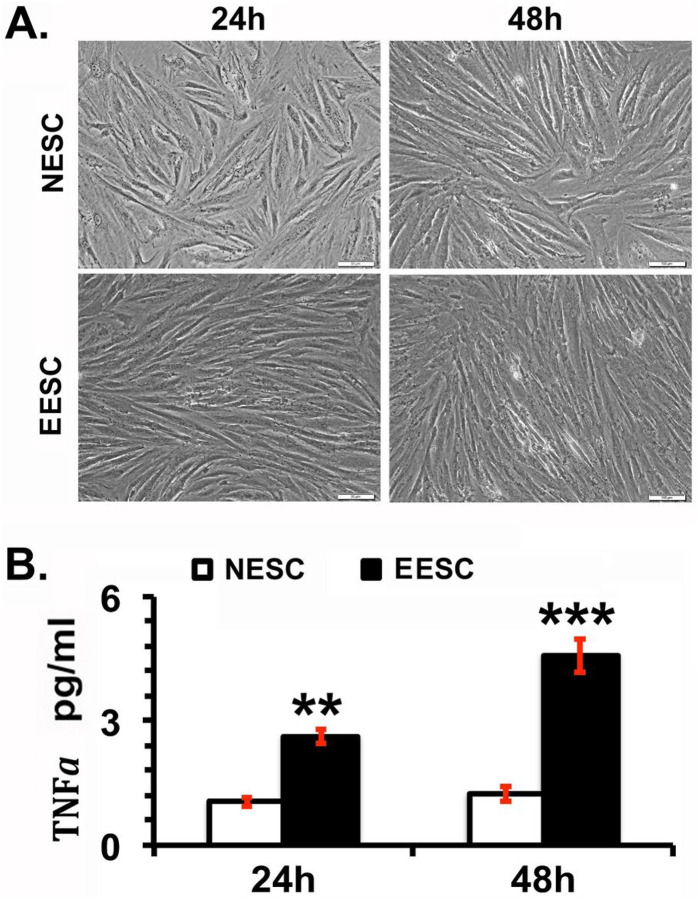
Analysis of morphological changes and pleiotropic cytokine tumor necrosis factor α (TNFα) expression in normal human endometrial stromal cells (NESCs) and cells derived from eutopic endometrium of endometriosis subjects (EESCs) *in vitro*. NESCs and EESCs were cultured as described in Material and Methods. A. The representative photographs showed the morphological changes in live cells taken under a phase contrast microscope at 200× magnification at 24 and 48 hours. B. Bar graph represents the concentrations of TNFα in the supernatants as mean ± SEM of results from three individual experiments (n=3). Post-hoc corrections for multiple comparisons were done by Newman-Keuls’ test. Star (*) represents significant differences (**P ≤ 0.01, ***P ≤ 0.001) between NESCs and EESCs groups.

We compared the secretion of TNFα in the culture media of serum-starved NESC and EESC *in vitro*. Although under phase contrast microscopy, there was no significant morphological difference observed between NESC and EESC at 24 and 48 hours (Fig. 1A), however, the concentration of TNFα was significantly higher in the culture media of EESCs compared to NESC at both 24 and 48 hours (Fig. 1B). Moreover, a higher TNFα secretion was observed after 48 hours in EESC media.

### miRNAs linked to inflammation are differentially expressed between NESC and EESC

**Figure 2:**
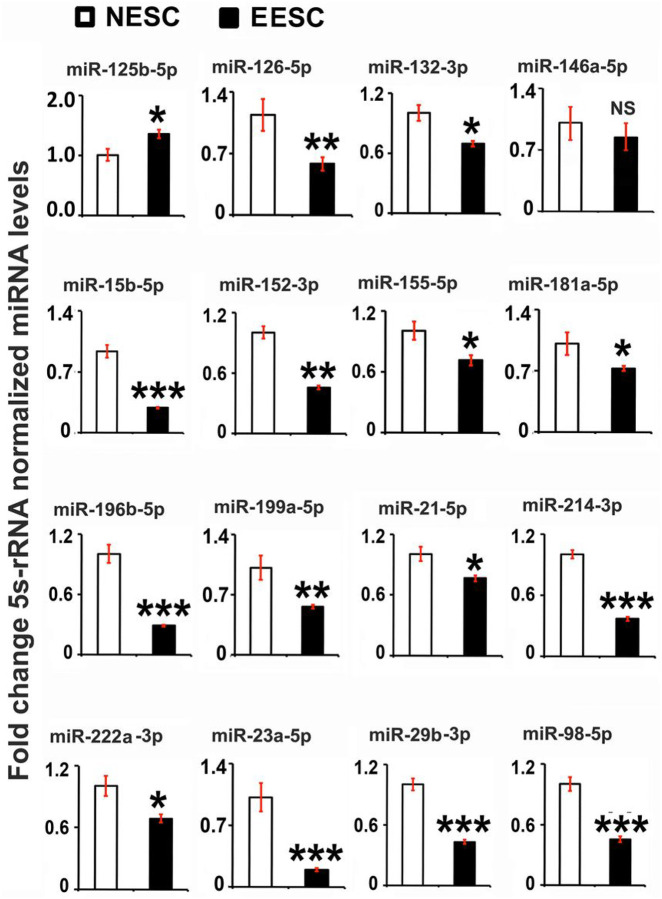
Analysis of selected miRNAs in normal human endometrial stromal cells (NESCs) and cells derived from eutopic endometrium of endometriosis subjects (EESCs) *in vitro*. Cells were cultured for 48 hours as described in Material and Methods. Total RNA was isolated, and selected miRNAs were analyzed by quantitative RT-PCR, normalized by 5S rRNA, and represented as fold changes between NESCs and EESCs. All bar graphs represent the mean ± SEM of results from three individual experiments (n=3). Unpaired Student’s t-test represents significant differences (*P ≤ 0.05,**P ≤ 0.01, ***P ≤ 0.001) between NESCs and EESCs groups. NS- No significant differences.

To better understand the correlative changes in the abundantly expressed miRNAs linked to the inflammation in endometriosis, the selected miRNAs (miR-125b-5p, miR-126-5p, miR-132-3p, miR-146a-5p, miR-15b-5p, miR-152-3p, miR-155-5p, miR-181a-5p, miR-196b-5p, miR199a-5p, miR-21-5p, miR-214-3p, miR-222a-3p, miR-23a-5p, miR-29b-3p, and miR-98-5p) [53] [54] [55] [56, 57] [58] [59] [60] [61] [62] [63] [62], were analyzed in NESCs and EESCs. The expression of the miRNA was measured at 48h in ESCs culture which conforms to the significant upregulation of TNFα secretion at 48h compared to 24h in EESCs. The expression level of miR-126-5p, miR-132-3p, miR-15b-5p, miR-152-3p, miR-155-5p, miR-181a-5p, miR-196b-5p, miR199a-5p, miR-21-5p, miR-214-3p, miR-222a-3p, miR-23a-5p, miR-29b-3p, and miR-98-5p were downregulated significantly (p < 0.05) in EESCs compared to NESCs, except for miRNA-125b-5p which showed a substantial upregulation in expression (Fig. 2). There was no significant changes in expression levels of miR-146a-5p,

#### TNFα treatment alters the expression of miRNAs and phosphorylation of PI3K, AKT, ERK and NF-κB in NESCs.

**Figure 3:**
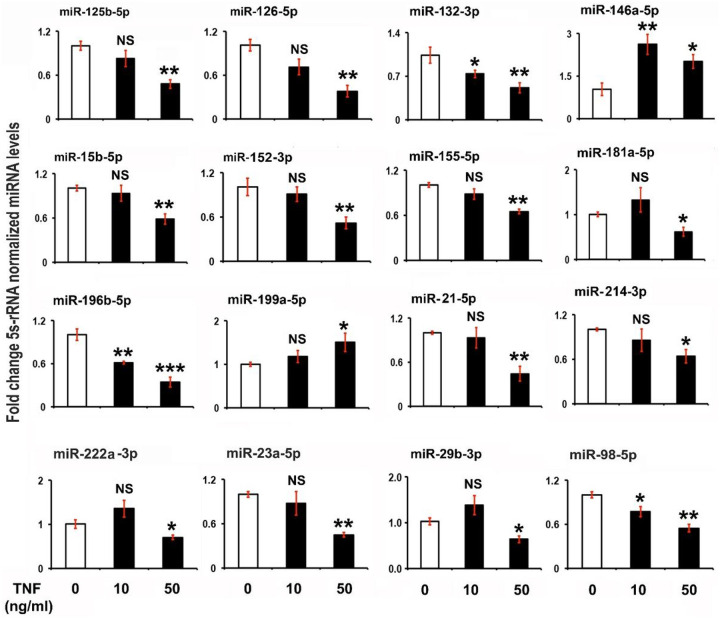
The effects of tumor necrosis factor α (TNFα) treatment on miRNA gene expression in normal human endometrial stromal cells (NESCs) *in vitro*. Cells were cultured and treated with TNFα for 24 hours as described in Material and Methods. Total RNA was isolated, and the expression of selected miRNAs was analyzed by quantitative RT-PCR, normalized for 5S rRNA concentrations, and represented as fold change of TNFα -treated cells compared to untreated NESCs. All bar graphs represent the mean ± SEM of results from three individual experiments (n=3). One-way ANOVA analysis of TNFα effects on miRNA expression in NESCs *in vitro* [miR-125b-5p, F(5,12)=30.51, P ≤ 0.001; miR-126-5p, F(5,12)=19.64, P ≤ 0.002; miR-132-3p, F(5,12)= 9.26, P ≤ 0.015; miR-146a-5p, F(5,12)=25.06, P ≤ 0.001; miR-15b-5p, F(5,12)=24.14, P ≤ 0.001; miR-152-3p, F(5,12)=56.94, P ≤ 0.0001; miR-155-5p, F(5,12)=63.34, P ≤ 0.001; miR-181a-5p, F(5,12)=10.19, P ≤ 0.012; miR-196b-5p, F(5,12)=82.03, P ≤ 0.0001; miR199a-5p, F(5,12)=5.22, P ≤ 0.05; miR-21-5p, F(5,12)=27.47, P ≤ 0.001; miR-214-3p, F(5,12)=9.88, P ≤ 0.013; miR-222a-3p, F(5,12)=25.57, P ≤ 0.001; miR-23a-5p, F(5,12)=27.90, P ≤ 0.001; miR-29b-3p, F(5,12)=19.89, P ≤ 0.001; and miR-98-5p, F(5,12)=11.67, P ≤ 0.01]. Post-hoc corrections for multiple comparisons were done by Newman-Keuls’ test. Star (*) represents significant differences (*P ≤ 0.05, **P ≤ 0.01, ***P ≤ 0.001) between NESCs and EESCs groups. NS- No significant differences.

To investigate the possible role of the increased level of the proinflammatory cytokine TNFα in ESCs with altered expression of miRNAs tied to the NF-κB and survival pathways, NESCs were treated with exogenous recombinant TNFα (10 and 50 ng/mL) for 24h *in vitro* [64] [24] [65]. The expression of miR-132-3p, miR-196b-5p, and miR-98-5p was downregulated whereas 146a-5p was significantly upregulated with TNFα treatment (10 and 50 ng/mL) after 24 hours (Fig. 3). Whereas low dose of TNFα (10 ng/mL) had no significant effect on the expression of any of the miRNAs mentioned here (miR-125b-5p, miR-126-5p, miR-15b-5p, miR-152-3p, miR-155-5p, miR-181a-5p, miR199a-5p, miR-21-5p, miR-214-3p, miR-222a-3p, miR-23a-5p, and miR-29b-3p (Fig. 3), a higher dose of TNFα (50 ng/mL for 24h) induced a strong inhibitory effect on all miRNAs except miR146a-5p and miR199a-5p, which was significantly upregulated (Fig. 3).

**Figure 4:**
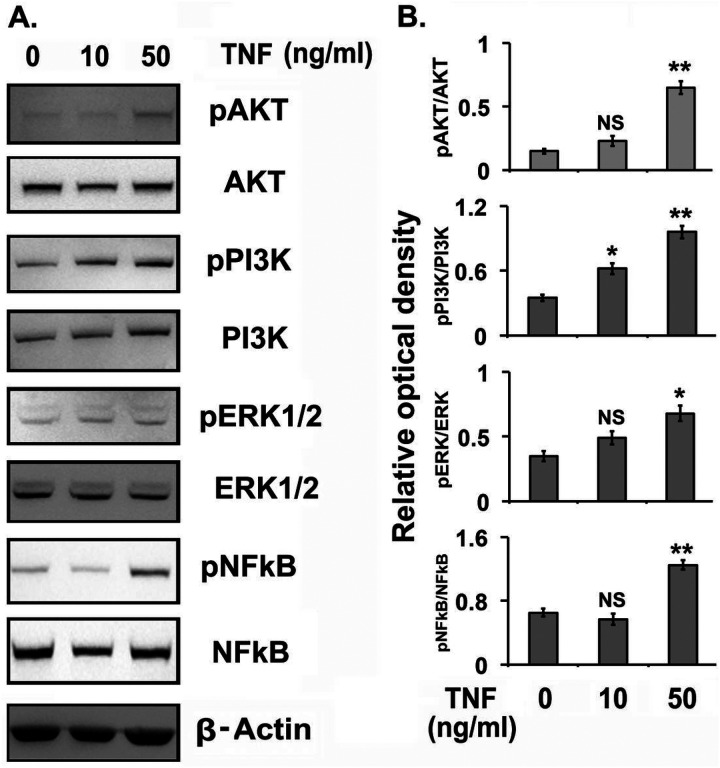
The effects of tumor necrosis factor α (TNFα) treatment on kinases in normal human endometrial stromal cells (NESCs) *in vitro*. Cells were cultured and treated with TNFα for 24 hours as described in Material and Methods. Total protein was isolated and the phosphorylation of AKT, PI3K, ERK1/2, and NF-κB were analyzed. A. Representative western blot (WBs) analysis for phospho- and total AKT, PI3K, ERK1/2, and NF-κB protein levels in NESCs treated with or without TNFα. B-actin was used as an internal constitutive control. B. The bar graphs represent the ratios of phospho-Akt, phosphor-PI3K, phospho-Erk1/2 and phospho-NF-κB protein levels normalized to total AKT, PI3K, ERK1/2, and NF-κB respectively. All bar graphs represent the mean ± SEM of results from three individual experiments (n=3). One-way ANOVA analysis of TNFα effects on pAKT/AKT [F(5,12)=170, P ≤ 0.0001], pPI3K/PI3K [F(5,12)=168.18, P ≤ 0.0001], pERK/ERK [F(5,12)=36.31, P ≤ 0.0001] and pNF-κB/NF-κB [F(5,12)=188.37, P ≤ 0.0001] expression in NESCs *in vitro*. Post-hoc corrections for multiple comparisons were done by Newman-Keuls’ test. Star (*) represents significant differences (*P ≤ 0.05, **P ≤ 0.01, ***P ≤ 0.001) between TNFα treated and untreated groups. NS- No significant differences.

TNFα is known to activate the PI3K/AKT pathway, which in turn activates the NF-κB signaling pathway [66] and are essential steps for proinflammatory gene expression. So we explored whether TNFα-treatment affects phosphorylation of PI3K, AKT, ERK, and NF-κB in NESC. As shown in Figs. 4A and 4B, the treatment of NESCs with TNFα at 50 ng/mL for 24h significantly increased the phosphorylation of PI3K, AKT, ERK, and NF-κB, whereas no significant effects on phosphorylation were noted at lower concentrations of TNFα except the phosphorylation of PI3K that is significantly higher in lower dose of TNFα.

### Curcumin treatment inhibits TNFα secretion and alters the expression of miRNAs

**Figure 5:**
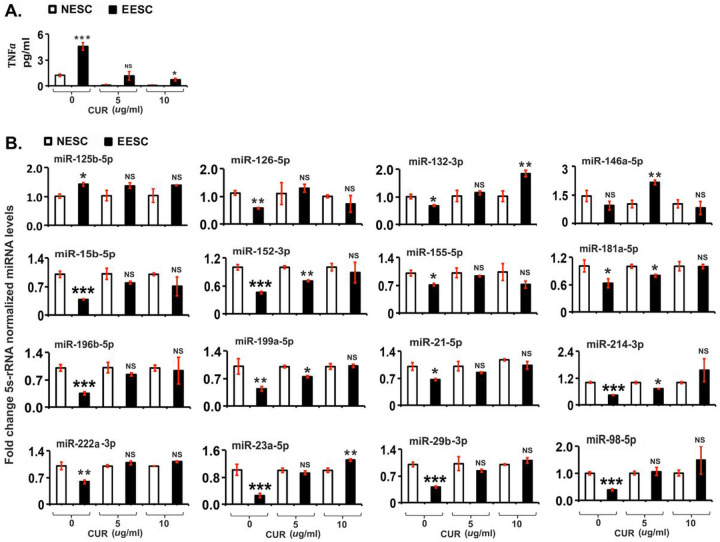
Effects of curcumin (CUR) on tumor necrosis factor α (TNFα) secretion and miRNA expression in human normal endometrial stromal cells (NESCs) and cells derived from eutopic endometrium of endometriosis (EESCs) subjects. Cells were treated with or without curcumin (CUR, 5ug/ml or 10ug/ml) for 48 hours as described in Material and Methods. A. Bar graph represents the concentrations of TNFα in the supernatants. B. Total RNA was isolated, and selected miRNAs were analyzed by quantitative RT-PCR, normalized over 5s rRNA, and represented as fold changes of the treated group over the untreated ones in both NESCs and EESCs All bar graphs represent the mean ± SEM of results from three individual experiments (n=3). One-way ANOVA analysis of CUR effects on TNFα [F(5,12)=99, P ≤ 0.0001], and miRNA expression [miR-125b-5p, F(5,12)=28, P ≤ 0.0001; miR-126-5p, F(5,12)=6.7, P ≤ 0.003; miR-132-3p, F(5,12)= 20.34, P ≤ 0.0001; miR-146a-5p, F(5,12)=12.2, P ≤ 0.0001; miR-15b-5p, F(5,12)=18.6, P ≤ 0.0001; miR-152-3p, F(5,12)=20.3, P ≤ 0.0001; miR-155-5p, F(5,12)=4.43, P ≤ 0.016; miR-181a-5p, F(5,12)=4.46, P ≤ 0.016; miR-196b-5p, F(5,12)=7.84, P ≤ 0.002; miR199a-5p, F(5,12)=36.95, P ≤ 0.0001; miR-21-5p, F(5,12)=23.05, P ≤ 0.0001; miR-214-3p, F(5,12)=9.86, P ≤ 0.001; miR-222a-3p, F(5,12)=42.03, P ≤ 0.0001; miR-23a-5p, F(5,12)=50.13, P ≤ 0.0001; miR-29b-3p, F(5,12)=26.7, P ≤ 0.0001; and miR-98-5p, F(5,12)=6.94, P ≤ 0.003]. in NESCs and EESCs *in vitro*. Post-hoc corrections for multiple comparisons were done by Newman-Keuls’ test. Star (*) represents significant differences (*P ≤ 0.05, **P ≤ 0.01, ***P ≤ 0.001) between NESCs and EESCs groups treated with CUR at 48 hours. NS- No significant differences.

To determine whether CUR treatment modulates the expression of miRNAs, EESCs, and NESCs were treated with different doses of CUR for 48hrs. To understand the mechanism better, TNFα secretion was analyzed post-CUR treatment. As shown in Fig. 5A, CUR treatment inhibited significantly the secretion (*p* ≤ 0.05) of TNFα in a dose-dependent manner in EESCs. In subsequent studies, the expression of selected miRNAs was analyzed under these experimental conditions. As shown in Fig. 5B, CUR treatment significantly promoted the expression of selected miRNAs, precisely at 5 μg/mL (miR-146a-5p) and at 10 μg/mL (miR-132-3p, miR-23a-5p) at 48 hours in EESC compared to NESC. Moreover, there is a downregulation of miRNA expression after 48 hours (miR-152-3p, miR-181a-5p, miR-199a-5p, miR-214-3p) at 5 μg/mL dose. However, there were no significant differences in the expression of most of the miRNAs in post-CUR-treated EESCs compared to NESCs at 48 hours (Fig. 5B).

## Discussion

The current findings suggest a new basis for understanding the mechanism of TNFα in the pathogenesis of endometriosis. The acute inflammatory response to TNFα is mediated by local dysregulation of miRNAs linked to NFkB-signaling pathways, thus contributing to the pathogenesis of the disease. It is well established that EESCs function differently in women with endometriosis compared with NESCs from disease-free women [67]. The current findings corroborate previously published data that EESCs have increased basal production of TNFα, which promotes a chronic inflammatory environment within the pelvis of these women [16] [68]. TNFα, along with other cytokines, is involved in the recruitment and activation of macrophages, neutrophils, eosinophils, basophils, monocytes, and NK-cells to the sites of endometriosis implants, enhancing EC proliferation and angiogenesis through increased production of VEGF and the adhesion of endometrium cells to the peritoneal cavity [69] [70] [71]. Moreover, elevated levels of TNFα in peritoneal fluid activate NFκB signaling along with other proinflammatory factors, which ultimately promote the proliferative and inflammatory characteristics of endometriosis [72] [73] [23] [24] [18] [19] [74] [22] [47].

The current study suggests that under basal conditions, increased production of TNFα in EESC is associated with dysregulation of the expression of selected miRNAs (miR-125-5p, miR-126-5p, miR-132-3p, miR-146a-5p, miR-15b-5p, miR-152-3p, miR-155-5p, miR-181a-5p, miR-196b-5p, miR199a-5p, miR-21-5p, miR-214-3p, miR-222a-3p, miR-23a-5p, miR-29b-3p, and miR-98-5p). It is well established that numerous miRNAs are altered in the eutopic and ectopic endometrium and lesions in women with endometriosis [53]; [40]; [27]; [33]; [28]; [59]; [30]; [36]; [37]; [38], [60]; [39]. Some downregulated miRNAs (miR-126-5p, miR-132-3p, miR-146a-5p, miR-15b-5p, miR-152-3p, miR-155-5p, miR-181a-5p, miR-196b-5p, miR199a-5p, miR-21-5p, miR-214-3p, miR-222a-3p, miR-23a-5p, miR-29b-3p, and miR-98-5p) are directly linked to the activation of inflammatory signaling molecule NF-κB which could be involved in the pathogenesis and progression of endometriosis [61]; [75]; [76]; [41].

Studies have demonstrated in endometriotic and other cells and tissues that miR-125b is involved in cell proliferation and migration [77]; [78], miR-126 suppresses inflammation, and reactive oxygen species (ROS) production [79]; [80]; [81], miR-15b-5p suppresses angiogenesis [82], [83]; [84], miR-152-5p acts as a tumor suppressor, inhibits cell proliferation and is downregulated in endometrial cancer [85]. miR-155 is involved in the attenuation of inflammatory pathways ([57]; [86], miR-196b is involved in self-renewal and proliferation [87]; [53], and miR-199a activates NFκB and inflammatory signaling pathways [88]; [89], [90]. Similarly, miR-21 plays an essential role in the resolution of inflammation by negative feedback of inflammatory pathways [91]; [33], miR-214-3p inhibits the proliferation, migration, and invasion of EC cells [58], miR-222-3p promotes proliferation, proangiogenesis, and invasion [92]; [93], [62], and miR23a is involved in local steroidogenesis-dependent inflammation and growth of ectopic ECs [94]; [95]. MicroRNA-29b is involved in a wide range of functions, including apoptosis, cell proliferation, invasion, adhesion, metabolism, and progression in endometrial cancer cells by direct regulation of PTEN [96]; [97] [98]. MiR-98 expression was found to be reduced in diseased EC tissues compared to normal tissues [99].

Our results further indicate that TNFα stimulation of NESCs dysregulates miRNA expression, phenocopying EESC and implying that these cells are TNFα responsive, with effects more pronounced at higher concentrations. These findings are consistent with previous studies indicating that a higher concentration of TNFα for a more extended exposure period promotes dysregulation of miRNAs expression, which may partly govern NF-κB-signaling molecules [64] [24] [65]. Moreover, we found that exogenous TNFα significantly downregulated several miRNAs in NESCs except for 146a-5p which was upregulated with TNFα treatment at both doses (10 and 50 ng/mL) and miR-199a-5p, which was upregulated at the higher dosage (50 ng/mL) after 24 hours. This apparent discrepancy could be a compensatory upregulation induced by a very high concentration of exogenous TNFα for an extended period or could be a part of a negative feedback loop reducing the impact of TNFα [90]. Furthermore, exogenous TNFα-dependent activation of PI3K/AKT/ERK1/2-signaling and NF-κB phosphorylation in NESCs suggest that TNFα may be an important cytokine contributing to the cascade of kinase signaling with dysregulation of miRNAs expression in ECs. Previous studies also established that TNFα-mediated activation of the PI3K/Akt and the NF-κB signaling pathway are essential steps for proinflammatory gene expression [66]. In endometriotic cells, NK-κB signaling is activated by TNFα [12] [100] [101] [47] and the aberrant activation of NF-κB signaling leads to chronic inflammation, increased cell proliferation, and survival of ECs in endometriosis [102] [23] [24] [18] [19] [20], [21] [22]. Previous studies have also demonstrated that the phosphorylation states of NF-κB signaling molecules, including IKKα, IKKβ, NF-κB, JNK, and STAT3, are higher in EESCs, which are involved in the downstream participation of various kinases linked to cytokine- and chemokine-specific membrane receptor complexes and adaptor proteins, that converge on NF-κB signaling pathway [16] [76] [103]. Thus, TNFα dependent dysregulation of miRNA expression in conjunction with altered phosphorylation of pPI3K/pAKT/pERK1/2/pNF-κB suggests a regulatory link that supports the idea of transformation of NESCs to a pathophysiological state similar to that of EESC.

Further studies revealed that CUR is a potent inhibitor of TNFα secretion from EESCs [16]. Moreover, our data showed that curcumin treatment could modulate TNFα mediated dysregulation of miRNAs in EESCs. The inhibitory effect of CUR is extended further to the attenuation of IKKα, IKKβ, and NF-κB [41] [16] [76] [104] [103]. IKKα and IKKβ are part of a multiprotein complex mediating the transcription of multiple chemokine and cytokine genes through I*k*β. Thus, our results are consistent with published reports showing that CUR has strong anti-inflammatory and antiangiogenic properties [16].

In conclusion, the current study provides new insights into how elevated levels of TNFα secretion are associated with aberrant expression of miRNAs in ECs, which subsequently alter phosphorylation of the proinflammatory molecule NF-κB and survival pathways. Moreover, CUR treatment modulates the dysregulation of miRNAs. Further studies are needed using genetic gain or loss-of-function models of individually selected miRNAs to pinpoint the pathophysiological effects of those miRNAs in inflammation during endometriosis. Based on the dynamic nature of miRNA expression combined with diverse actions and multiple targets of NF-κB-signaling molecules, we believe that an NF-κB-miRNA feedback loop should be considered in inflammatory responses and initiation, progression, and development of endometriosis. Moreover, understanding the intersection of NF-κB signaling molecules and miRNA regulatory networks may offer opportunities for pharmacological exploitation and personalized treatment for endometriosis pain management.

## Figures and Tables

**Figure 6:**
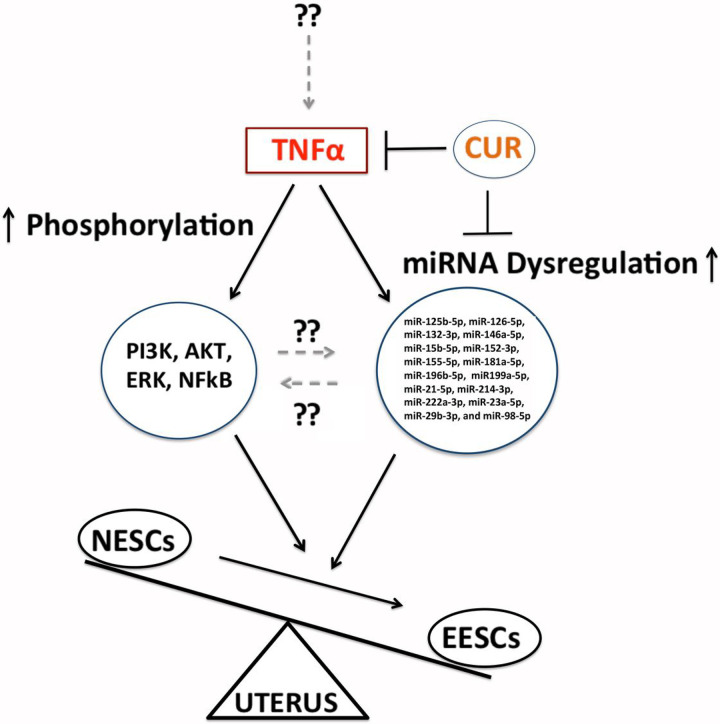
A schematic model showing the effects of curcumin (CUR) on tumor necrosis factor α (TNFα) secretion, activation of PI3K/AKT/ERK1/2-signaling and NF-κB phosphorylation with dysregulation of miRNAs expression in endometrial stromal cells. CUR: curcumin; ESCs: endometrial stromal cells; N:Normal; E: Endometriotic; NF-κB: nuclear factor κ-light-chain-enhancer of activated B; arrow represents promotion and blunt arrow represents inhibition,.

**Table 1:** List of antibodies used for Western blot (WB) analysis.

Peptide/Protein target	Name of antibody	Name of the company providing the antibody	Species raised (Monoclonal or Polyclonal)	Research Resource Identifier (RRID)	Dilution used
Phospho nuclear factor kappa-light-chain-enhancer of activated B cells (pNFκB)	Anti-Phospho NFkB (pNFκB)	Cell Signaling, Beverly, MA, USA	Rabbit Monoclonal	AB_331284	1:1000
Nuclear factor kappa-light-chain-enhancer of activated B cells (NFκB)	Anti-NFκB (NFκB)	Cell Signaling, Beverly, MA, USA	Rabbit monoclonal	AB_10859369	1:1000
pErk1/2	Anti-pErk1/2	Cell Signaling, Beverly, MA, USA	Mouse monoclonal	AB_2297442	1:1000
Total Erk1/2	Anti-Total Erk1/2	Cell Signaling, Beverly, MA, USA	Rabbit monoclonal	AB_331775	1:1000
pAkt	Anti-pAkt	Cell Signaling, Beverly, MA, USA	Rabbit polyclonal	AB_329825	1:1000
Total Akt	Anti-Total Akt	Cell Signaling, Beverly, MA, USA	Rabbit polyclonal	AB_329827	1:1000
pPI3K	Anti-pPI3K	Cell Signaling, Beverly, MA, USA	Rabbit polyclonal	AB_659940	1:1000
Total PI3K	Anti-Total PI3K	Cell Signaling, Beverly, MA, USA	Rabbit monoclonal	AB_659889	1:1000
β-Actin	Anti-Beta-Actin	Cell Signaling, Beverly, MA, USA	Rabbit monoclonal	AB_330288	1:1000

## Data Availability

We confirm that the manuscript has been read and approved by all named authors and that there are no other persons who satisfied the criteria for authorship but are not listed. We further confirm that the order of authors listed in the manuscript has been approved by all of us. The authors confirm that the data supporting the findings of this study are available within the article. Therefore, any other declaration is “not applicable”.
